# A sequence-dependent exonuclease activity from *Tetrahymena thermophila*

**DOI:** 10.1186/1471-2091-11-45

**Published:** 2010-11-16

**Authors:** Hui-I Kao Tom, Carol W Greider

**Affiliations:** 1 Department of Molecular Biology and Genetics, The Johns Hopkins University, School of Medicine, Baltimore, Maryland 21205, USA

## Abstract

**Background:**

Telomere function requires a highly conserved G rich 3'- overhang. This structure is formed by 5'-resection of the C-rich telomere strand. However, while many nucleases have been suggested to play a role in processing, it is not yet clear which nucleases carry out this 5'-resection.

**Results:**

We used biochemical purification to identify a sequence-dependent exonuclease activity in *Tetrahymena thermophila *cell extracts. The nuclease activity showed specificity for 5'-ends containing AA or AC sequences, unlike Exo1, which showed sequence-independent cleavage. The *Tetrahymena *nuclease was active on both phosphorylated and unphosphorylated substrates whereas Exo1 requires a 5'-phosphate for cleavage.

**Conclusions:**

The specificities of the enzyme indicate that this novel *Tetrahymena *exonuclease is distinct from Exo1 and has properties required for 3'-overhang formations at telomeres.

## Background

Functional telomeres are essential for cell survival. Telomeres distinguish natural chromosome ends from DNA breaks. When telomere function is lost, cells arrest and undergo senescence or apoptosis [1-6]. Normal DNA replication causes telomere shortening, which is counterbalanced by the extension activity of telomerase [7]. The majority of cancers have telomerase activity, and this activity is needed for ongoing division of tumor cells. In somatic cells, telomerase activity is absent or low. When cell division occurs faster than the ability of telomerase to elongate telomeres, the short telomeres limit cell replicative capacity [1,8]. This limited cell division underlies diseases of tissue renewal such as dyskeratosis congenita, aplastic anemia, and pulmonary fibrosis [9-13]. Thus, telomere length regulation plays a pivotal role in cancer and in age-related degenerative disease.

Both telomeric DNA structure and telomerase play a role in establishing telomere length and end protection. Telomeres have a G-rich 3'-overhang that was first discovered in ciliates [14] and is a conserved feature of telomeres in all eukaryotes [14-21]. This 3'-overhang is the binding site for essential telomere proteins, such as Cdc13 in *Saccharomyces cerevisiae *and Pot1 in mammals and other species. Loss of the overhangs due to disruption of the telomere complex, termed shelterin, leads to telomere-induced DNA damage response and chromosome end-to-end fusion [22,23]. In addition to maintenance of DNA ends, the 3'-overhang is essential for telomerase accessibility. Telomerase recognizes and elongates single-stranded DNA substrates, but not double-stranded substrates [24]. Thus, the processing of blunt ends, generated by replication, is needed for telomerase to elongate telomeres. *In vivo*, blocking 3'-overhang formation in *S. cerevisiae *blocks the ability of telomerase to elongate a telomere seed sequence [25].

3'-overhang formation is an active process. In *S. cerevisiae *and mice, generation of the 3'-overhang occurs in the absence of telomerase [26,27]. In *Tetrahymena*, a conditional telomerase protein component (TERT) knockout cell line showed that telomerase depletion had relatively little effects on absolute G overhang length, indicating C-strand degradation, not telomerase elongation of the G-rich strand, must generate the overhang structures [28]. Inhibition of Cdk1 blocks C-rich strand resection, thereby inhibiting 3'-overhang production [25,29]. These data suggest that there is a nuclease regulated by Cdk1 that processes the telomeres prior to elongation by telomerase. Additional evidence for an active processing enzyme comes from the fact that both ends of a chromosome have similar overhang structures. Replication of a chromosome to the very end is predicted to generate one chromosome end with a 3'-overhang and one with a blunt end [30]. However, chromosomes in ciliates as well as in yeast and mammals have 3'-overhangs on both ends, [16,18,31-33], implying at least one end must be processed to generate the overhang structure.

Finally, the very precise structure of the telomere overhangs implies that it is formed by a regulated process. The length and sequence of the 3'-overhangs in *Oxytricha *and *Tetrahymena *are surprisingly homogeneous [14,16]. In *Oxytricha*, there is a precise overhang of two 8 nucleotide 5'-TTTTGGGG-3' repeats, whereas in *Tetrahymena*, the overhangs are either 14-15 or 20-21 nucleotides in length, and the majority of 5'-strands end in 3'-CCAACC-5', or 3'-CAACCC-5' [16,28]. In mammals, this overhang length is also conserved [34]. This functional and structural evidence argues for the existence of a sequence-specific nuclease that processes telomeres. To investigate the role of 3'-overhang processing, we set out to identify a telomere-specific nuclease activity. We chose *Tetrahymena *to look for processing activity because it has uniform telomere repeat sequence, a precise 3'-overhang structure, and a history as an excellent biochemical source for telomere-processing factors. Here we describe a sequence-dependent 5' to 3' exonuclease activity from *Tetrahymena *that processes the C-strand of telomere substrates *in vitro*.

## Results

**Figure 1:**
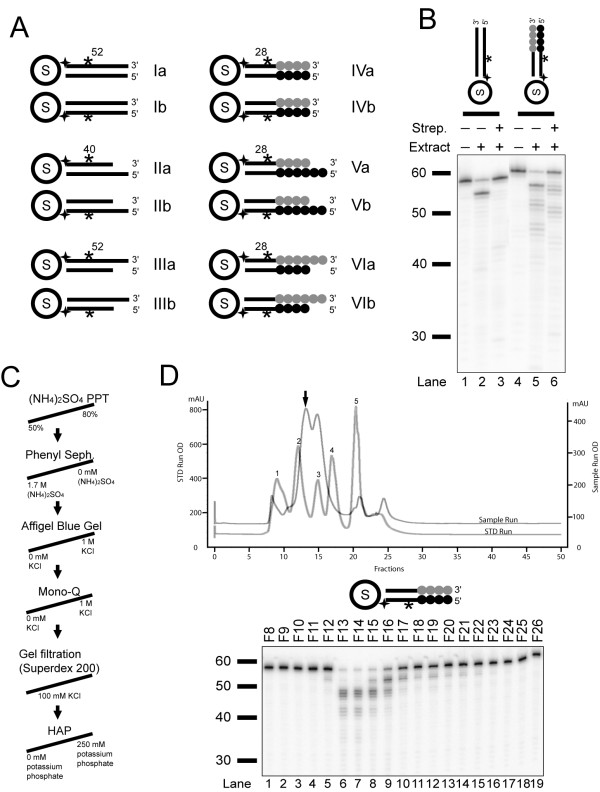
**Design of oligonucleotide substrates and detection of a telomere-processing nuclease activity**. (A) Schematic representation of the substrate structures utilized in detecting the telomere-processing nuclease in *Tetrahymena *extracts. Solid lines represent non-telomeric sequences, and the numbers indicate lengths of non-telomeric base pairs in the duplex region. Each gray circle represents a TTGGGG repeat unit, and each black circle is a complementary CCCCAA repeat unit. S inside a circle represents the streptavidin protein. The stars indicate the approximate location of radiolabels, and 4-prong symbols are biotinylation sites. The annealing components were: Ia-S1*:S18; Ib-S1:S18*; IIa-S2*:S18; IIb-S2:S18*; IIIa-S1*:S19; IIIb-S1:S19*; IVa-S3*:S20; IVb-S3:S20*; Va-S3*:S21; Vb-S3:S21*; VIa-S4*:S20; VIb-S4:S20*. (B) *Tetrahymena *crude cell extracts were assayed with Ib and IVb and with/without streptavidin. ~ 1.2 μg total proteins from the extracts were incubated in each reaction for 15 min. (C) Nuclease purification scheme is presented. (D) The nuclease is 100 kDa in size. Chromatographs of gel filtration runs for standards and nuclease samples are shown in a superimposed graph. The peaks of standard proteins are labeled with numbers: peak 1-bovine thyroglobulin, 670,000; peak 2-bovine γ-globulin, 158,000; peak 3-chicken ovalbumin, 44,000; peak 4-horse myoglobin, 17,000; peak 5-vitamin B12, 1,350. The arrow indicates where the nuclease activity was eluted off the column. The apparent molecular weight was calculated as described under Methods. The activity corresponding to the fractions were indicated in the second panel with ~ 300-600 ngs protein per reaction.

To identify an overhang-processing nuclease, we assayed cell extracts from *Tetrahymena *[7]. We designed specific duplex oligonucleotide substrates to detect a telomere-specific nuclease activity (Figure 1A). The duplex oligonucleotide substrate contains specific non-telomeric sequences with a biotin moiety that binds to streptavidin to block non-specific degradation from one end (Additional file 1, supplementary Figure S1 and Figure 1B, compare *lanes 2 to 3 *and *lanes 5 to 6*). On the other end, the substrates contained either telomere repeats (5'-GGGGTT-3') or non-telomeric sequence. We tested substrates that were either blunt-ended, or with a 5'- or a 3'-overhang. To distinguish size changes of the substrates on each strand, we labeled each strand of the substrate separately in order to monitor processing occurred on the corresponding strand (Figure 1A).

### Identification of a nuclease activity from *Tetrahymena *crude extracts with preference for telomere substrates

We initially incubated the non-telomeric and telomeric blunt-ended substrates with crude *Tetrahymena *cell extracts (Figure 1B) and analyzed processing by gel electrophoresis and autoradiography. We observed a nuclease activity that degraded the telomeric substrate to a greater degree than the non-telomeric substrate (compare *lanes 3 *to *6*). This activity was robust, strand-specific, and sequence-dependent. The nuclease cleaved the 5'-strand of a blunt-ended substrate containing telomere repeats.

To further characterize this nuclease activity, we performed biochemical purification. Extracts were precipitated with ammonium sulfate, and then enriched on several fast protein liquid chromatography (FPLC) columns in the following order: Phenyl Sepharose, Affi-Gel Blue Gel (AGB), Mono-Q, Gel Filtration-Superdex 200, and Hydroxyapatite (HAP) (Figure 1C). The final active fractions were concentrated by a Mono-Q or a Q-spin column. The activity of the fractions from each step of the purification was analyzed, and the purification was monitored by Bradford protein assay and SDS-PAGE (Additional file 2, supplementary Figure S2). The final Q concentration step showed that the nuclease activity was highly enriched in three fractions (Additional file 2, supplementary Figure S2B, *lanes 4-6*). The active fraction contained low amount of protein, which could only be visualized on a silver-stained gel (Additional file 2, supplementary Figure S2A, *lane 5*). The gel filtration step of the purification indicated the active nuclease was approximately 100 kDa in size (Figure 1D).

**Figure 2:**
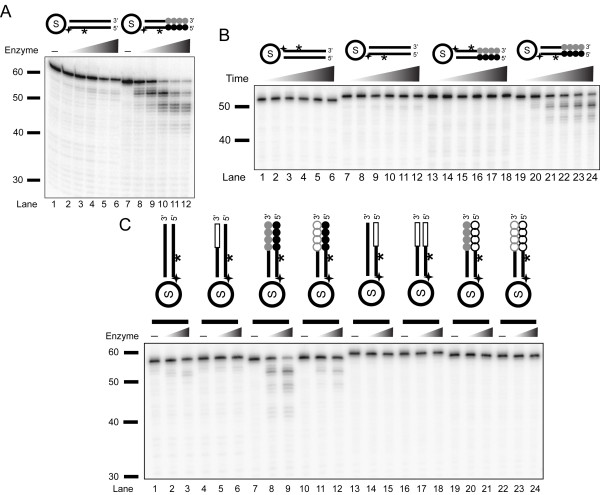
**Enzyme titration and time course of purified telomere-specific nuclease that is a DNase**. The triangles indicate increasing enzyme concentrations or time. (A) An enzyme titration of the purified telomeric nuclease (0, 0.95, 1.9, 4.75, 9.5, and 15.8 ng) is shown with substrates Ib and IVb. The reactions were incubated for 20 min. (B) Time course (0, 5, 10, 15, 20, and 30 min.) of purified telomeric nuclease at 4.75 ng per reaction with substrates Ia, Ib, IVa, and IVb is shown. (C) Cleavage of DNA but not RNA. Boxed sequences are non-telomeric ribonucleotides. Open gray circles are GGGGUU ribonucleotide repeat units, and open black circles are AACCCC ribonucleotide repeat units. Substrates (left to right) are Ib, M1:S18*, IVb, M2:S20*, S1:M3*, M1:M3*, S3:M4*, and M2:M4*.

After obtaining highly-active fractions, we characterized the specificity of the enzyme; both enzyme titration and time-course assays showed significant preference for telomeric over non-telomeric substrates (Figures 2A and 2B). The telomere repeat-containing substrates were efficiently processed by the nuclease (Figure 2A*, lanes 7-12 *and Figure 2B*, lanes 19-24*) throughout the incubation time while the non-telomeric and 5'-strand labeled substrates were only minimally degraded (Figure 2A,* lanes 1-6 *and Figure 2B*, lanes 7-12*). Cleavage was specific to telomeric 5'-strands: the substrates that were labeled on the 3'-strand showed no degradation by the nuclease (Figure 2B,* lanes 1-6 and 13-18*). Thus, processing of the blunt substrates by this nuclease leads to 3'-overhang formation.

### The *Tetrahymena *nuclease is a DNA exonuclease

We examined whether the *Tetrahymena *nuclease could cleave RNA substrates. We tested both non-telomeric and telomeric DNA (Figure 2C, *lanes 1-12*) and the corresponding RNA (*lanes 13-24*) in substrate structures as either heteroduplex DNA/RNA or homoduplex DNA/DNA or RNA/RNA. The nuclease cleaved the telomeric DNA substrates in a DNA/DNA homoduplex (*lanes 7-9*), but showed a lower level of cleavage activity on the telomeric DNA/RNA in a heteroduplex structure where the C strand was the DNA strand (*lanes 10-12*). No cleavage activity was observed when the C strand was made of RNA in either RNA/RNA or RNA/DNA duplexes (*lanes 13-24*). Thus, we conclude that the *Tetrahymena *nuclease is a DNase, specific to cleaving DNA linkages.

**Figure 3:**
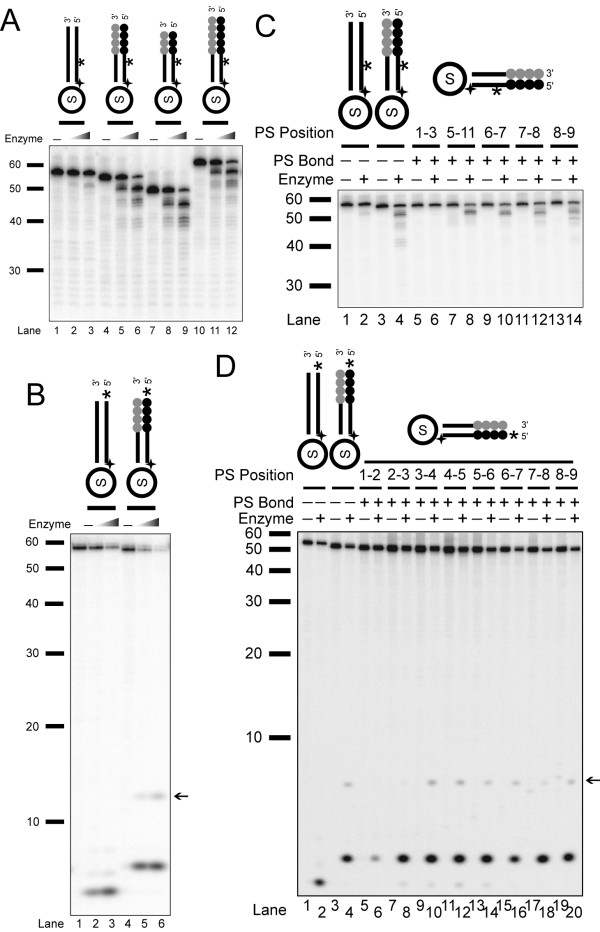
***Tetrahymena *nuclease is an exonuclease**. (A) Enzyme titrations (0, 0.46, and 2.3 ng) were incubated for 20 min. (on a 15% urea gel). Substrates (left to right) are Ib, IVb, S16:S33*, and S17:S34*. (B) Enzyme titration (0, 0.95, and 9.5 ng) of enriched nuclease with end-labeled non-telomeric, S1:S18 (*lanes 1-3*), and telomeric, S3:S20 (*lanes 4-6*), substrates (on a 15% urea gel). Arrow indicates an unique cleavage product of this nuclease. (C) Internally-labeled phosphorothioate substrates (with 0.9 ng of enzyme and substrates Ib, IVb, and various blockage locations on IVb, S3:S20) and (D) end-labeled phosphorothioate substrates (with 20 ng of enzyme and substrates S1:S18, S3:S20, and various single-bond blockage on S3:S20) are shown. Arrow indicates an unique cleavage product of this nuclease that is blocked by terminal phosphorothioate. Both assays were resolved on 15% urea gels.

To determine whether the repeat length affected nuclease cleavage activity, we examined the consequence of varying the repeat numbers in the telomeric substrates (Figure 3A). We observed substantial cleavage at similar level on 3-repeat, 4-repeat, and 5-repeat oligonucleotide, indicating that the cleavage by this *Tetrahymena *nuclease is not affected by telomeric repeat length.

The lack of length dependence suggested that the enzyme may be degrading the substrates from the end, like an exonuclease. Exonuclease activity removes a mononucleotide from free DNA ends while an endonuclease removes oligonucleotide at each cleavage event [35]. To examine the type of degradation this nuclease exhibits, we used end-labeled substrates rather than the internally-labeled substrates described above (Figure 3B). With these end-labeled phosphorylated substrates, we observed cleavage on both non-telomeric and telomeric substrates (Figure 3B, *lanes 2-3 *and *5-6*), and the cleavage product was mononucleotide. The decreased specificity for telomere repeats was due to the addition of the phosphate on the 5' strand as described in detail below. Because the first nucleotides on the non-telomeric and telomeric substrates were different, the mononucleotide products exhibited different mobility on a gel. This data suggested that *Tetrahymena *nuclease is an exonuclease.

To further examine the potential exonuclease activity of the *Tetrahymena *enzyme, we used oligonucleotides with specific bonds containing phosphorothioate linkage modification. This modification blocks cleavage activity of nucleases. We first tested the internally-labeled substrates that each contained one to several adjacent phosphorothioate linkages at positions 1-3, 5-11, 6-7, 7-8, and 8-9 (counting from the 5' terminus of the substrate) (Figure 3C). While the unmodified non-telomeric and telomeric substrates behaved as expected, we saw complete blockage of nuclease activity when there were phosphorothioate linkages at position 1-3 (*lanes 5-6*). When phosphorothioate linkages were placed at more internal positions, we saw significant cleavage that did not extend as far into the DNA as the unmodified substrates, indicating cleavage only occurred up to the blockage sites (*lanes 7-14*). To further examine the exonuclease activity, we synthesized substrates with individual bonds blocked with phosphorothioate modification and labeled at the 5'-end (Figure 3D). We tested these 5'-labeled substrates with the *Tetrahymena *nuclease. Blockage between nucleotide positions 1-2 significantly reduced the cleavage by the nuclease (*lanes 5-6*) whereas terminal cleavage still occurred on all other substrates (*lanes 7-20*). We concluded that the *Tetrahymena *nuclease activity we isolated is an exonuclease.

When using end-labeled substrates, we also detected a band that appeared to be larger than a mononucleotide (indicated by an arrow, Figures 3B and 3D). While the position of this band first suggested that it might be an endonucleolytic cleavage product, it was blocked by the phosphorothioate at the 1-2 position. We tried to map the size of this product, but its mobility varied depending on the acrylamide concentration, and it did not line up with either 3'-phosphate or 5'-phosphate marker ladders (data not shown). We conclude that this may be a modified nucleotide given that its generation is blocked by the terminal phosphorothioate linkage.

### The *Tetrahymena *nuclease only cleaves specific permutations of the 5'-telomeric C-rich strand

**Figure 4:**
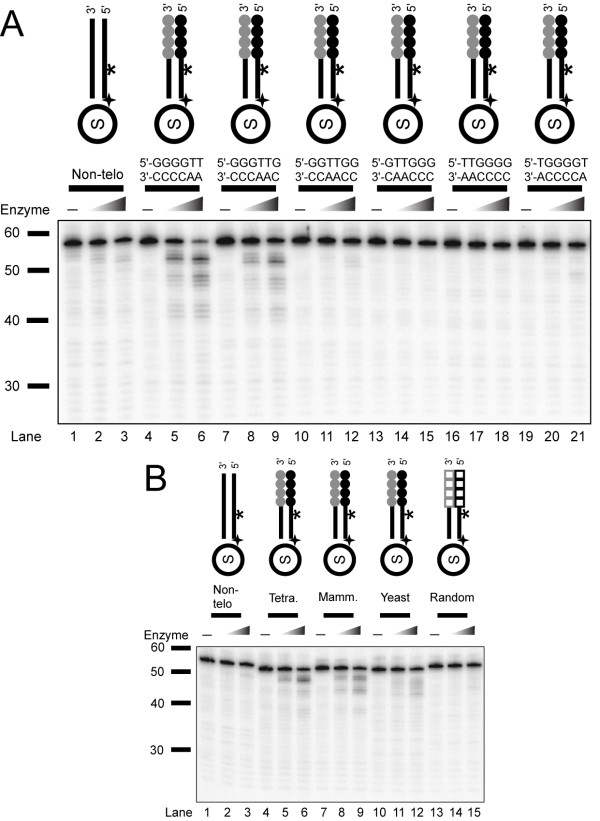
**Cleavage and substrate specificity of telomere-specific nuclease activity**. An enzyme titration (represented by triangles) using, 0, 0.46, and 2.3 ng (in A) or 0, 0.23, 0.93 ng (in B) was performed for a 20-min. incubation time. (A) Substrates (left to right) are with various repeat permutations: Ib, IVb, S9:S26*, S10:S27*, S11:S28*, S12:S29*, and S13:S30* (on a 15% urea gel). (B) Substrates (left to right) are Ib, IVb-*Tetrahymena *GGGGTT repeat, S14:S31*-mammal AGGGTT repeat, S15:S32*-yeast G_1-3_T repeat, and S8:S25*-GACGAT random repeat (on a 15% urea gel).

To further examine the sequence specificity of the exonuclease activity, we examined all the permutations of the *Tetrahymena *6-nucleotide telomeric repeat sequence. Surprisingly, there was significantly more cleavage of telomeric substrates with sequences ending in 5'-AA or 5'-CA while other permutations were not cleaved (Figure 4A). We also tested telomeric repeat sequences from human (5'-AACCCT-3') and yeast (5'-AC_1-3_-3') and observed cleavage on both of these substrates (Figure 4B). As a negative control, we tested a random non-telomeric 6-nucleotide repeat (5'-ATCGTC-3') and found no significant degradation (*lanes 13-15*).

The specificity for certain permutations of the CCCCAA repeat suggested that there may be an end-nucleotide sequence preference. Many exonucleases have been biochemically characterized and have been grouped based on their processing polarity, domain similarity, and substrate specificity, such as structure-specific 5' nucleases, 3'-5' exonucleases, metallo-beta-lactamase family, and single-stranded-specific nucleases (reviewed in [36-40]). None of these exonucleases that have been previously characterized show sequences specificity. Thus, we sought to characterize the sequence specificity of this *Tetrahymena *exonuclease in detail.

**Figure 5:**
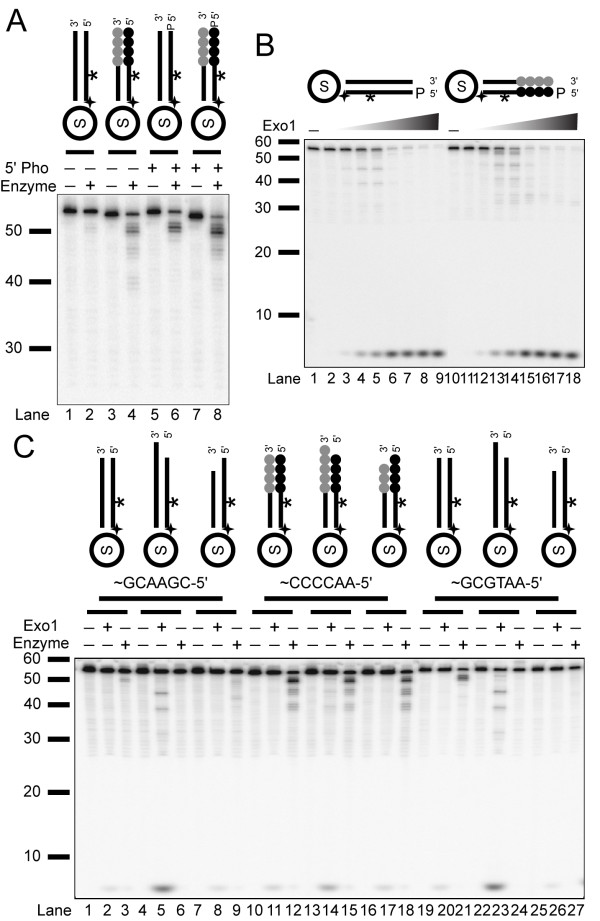
**The *Tetrahymena *nuclease shows sequence specificity and higher activity with substrates containing 5'- phosphates**. (A) Activity of enriched *Tetrahymena *nuclease (30 ng) on non-telomeric (Ib) and telomeric (IVb) substrates with and without 5'-phosphate is shown (*lanes 5-8 *and *lanes 1-4*, respectively) (20 min reactions). (B) Enzyme titration (0, 0.01, 0.1, 0.5, 1, 5, 10, 50, 100 fmol) of Exo1 purified protein with phosphorylated internally-labeled substrates S1:S18* (*lanes 1-9*) and S3:S20* (*lanes 10-18*) (20 min reactions on a 15% urea gel). (C) Activity of *Tetrahymena *nuclease (30 ng) and Exo1 (1 fmol or 0.04 ng) was assayed. Unique sequence (*lanes 1-9*), telomeric repeats (*lanes 10-18*), and AA-ending unique sequence (*lanes 19-27*) in either blunt, 3'-overhang, or 5'-overhang structures (10 min reactions on a 10% urea gel) are tested. Substrates (left to right) are: Ib, S54:S18*, S55:S18*, IVb, S34:S20*, S33:S20*, S52:S74*, S56:S74*, and S55:S74*.

Since our nuclease is an exonuclease, to examine the sequence specificity, we compared the activity of the *Tetrahymena *exonuclease to the activity of the well-characterized Exo1 that has been implicated in telomere processing. Exo1 has a strong preference for cleavage of DNA with a 5'-phosphate [41]. Thus, we first compared the cleavage of 5'-phosphorylated and unphosphorylated substrates with the purified *Tetrahymena *nuclease (Figure 5A). Both phosphorylated and unphosphorylated telomeric substrates were cleaved to a similar extent by the *Tetrahymena *nuclease (compare *lanes 8 *to *4*). There was also an increase in cleavage activity on the 5'-phosphorylated, non-telomeric substrate. While this non-telomeric cleavage on 5'-phosphorylated substrate was less extensive than cleavage on the telomeric substrate, this data implies a reduced specificity for telomeric substrates on 5'-phosphorylated DNA. We next utilized human Exo1 in cleavage assays to compare the biochemical properties of our *Tetrahymena *nuclease. For convenience, whenever Exo1 activity is mentioned in this study, it refers to human Exo1. Enzyme titration of Exo1 with phosphorylated substrates showed that it robustly cleaves DNA from 5'-end into the substrate up to the internal labeling site, releasing the internally labeled nucleotide that runs at the bottom of the gel (Figure 5B). However, Exo1 did not show any preference for telomeric versus non-telomeric substrates (compare *lanes 1-9 *to *10-18*).

Besides cleavage on single-stranded DNA, Exo1 also exhibits structural preferences on certain substrates. As an exonuclease, Exo1 prefers 5'-recessed structures, and as an endonuclease, it cleaves flapped structures [41,42]. Because of these structural preferences for Exo1, we tested the specificities of these two nucleases with substrates that had different end structures (Figure 5C). We designed telomeric, and non-telomeric substrates ending in either 5'-CGAA or 5'-AATG that were either blunt, or had a 3'- or 5'-overhang. Using non-phosphorylated substrates, all of the telomeric substrates were actively cleaved by the *Tetrahymena *nuclease (*lanes 12, 15 *and* 18*). The Exo1 digestions showed a preference for substrates that contained a 3' overhang (*lanes 5*, *14* and *23*) as described previously [41]. We also examined the activities of both enzymes on phosphorylated substrates and found similar results, although the level of Exo1 cleavage was higher (data not shown). Since there was no preference for the different structures tested with the *Tetrahymena *exonuclease, we concluded that this enzyme does not show the structure-dependent activity of Exo1.

### *Tetrahymena *exonuclease shows end-nucleotide specificity

Because the *Tetrahymena *exonuclease showed a preference for AA and CA terminal nucleotides on nonphosphorylated telomeric substrates (see Figure 4A), we next ask whether end-sequence specificity is preserved in the presence of 5'-phosphorylated substrates. We examined the six different permutations of the telomere repeat using both phosphorylated and nonphosphorylated telomeric substrates. The *Tetrahymena *nuclease showed sequence preference for AA and CA ending substrates (Additional file 3, supplementary Figure S3), while Exo1 did not show significant sequence specificity.

**Figure 6:**
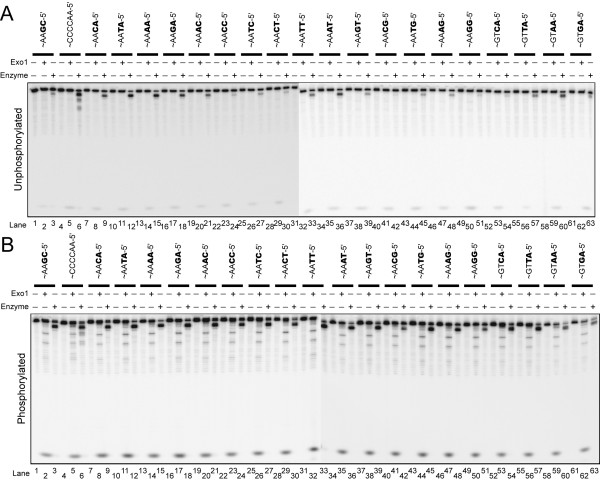
**Enriched *Tetrahymena *nuclease shows sequence-dependant cleavage**. Activity of *Tetrahymena *nuclease (30 ng) and Exo1 (1 fmol or 0.04 ng) with various unique sequences that end in all combinations of two terminal nucleotides, either unphosphorylated (A) or phosphorylated (B) were tested. Reactions were performed for 20 min and resolved on 10% urea denaturing polyacrylamide gels. All assays were run and performed on the same day, and the time for exposure to phosphorimager screens was the same. Substrates used are: S1:S18* (*lanes 1-3*), S3:S20* (*lanes 4-6*), S35:S57* (*lanes 7-9*), S36:S58* (*lanes 10-12*), S37:S59* (*lanes 13-15*), S38:S60* (*lanes 16-18*), S39:S61* (*lanes 19-21*), S40:S62* (*lanes 22-24*), S41:S63* (*lanes 25-27*), S42:S64* (*lanes 28-30*), S43:S65* (*lanes 31-33*), S44:S66* (*lane 34-36*), S45:S67* (*lanes 37-39*), S46:S68* (*lanes 40-42*), S47:S69* (*lanes 43-45*), S48:S70* (*lanes 46-48*), S49:S71* (*lanes 49-51*), S50:S72* (*lanes 52-54*), S51:S73* (*lanes 55-57*), S52:S74* (*lanes 58-60*), S53:S75* (*lanes 61-63*).

We next tested a series of non-telomeric substrates with all possible permutations of the 5'-terminal two nucleotides (Figure 6). The substrates were all internally-labeled. When testing unphosphorylated substrates (Figure 6A), there was some cleavage of all substrates, except when G was the terminal nucleotide. There was a clear preference for substrates ending with an A residue. The order of cleavage preference for the terminal nucleotide was A>T>C>G (*lanes 1-3 *and *7-51*). However, the cleavage pattern on these non-telomeric substrates was limited to terminal nucleotides compared to the more extensive cleavage of the telomeric substrate (compare *lanes 4-6 *to *22-24*). This difference in the extent of cleavage may be due to the specific nucleotide that is exposed next or may be influenced by contacts with internal nucleotides in the telomere repeats. The possibility of contacts with internal nucleotides could also explain the low cleavage observed on yeast repeat sequences ending in 5'-AC when compared to the *Tetrahymena *repeat permutation ending in 5'-AC (Figure 4). Incubation of the *Tetrahymena *exonuclease with the same set of sequences that were 5'-phosphorylated showed a similar trend of sequence specificity (Figure 6B). Exo1 was tested along side the *Tetrahymena *exonuclease, and it cleaved all phosphorylated substrates well. However, Exo1 did not show any specificity for the terminal nucleotide sequences (Figure 6B), note the similar intensity of the mononucleotide band in every 3^rd ^lane on the gel. Unphosphorylated substrates were also cleaved by Exo1 without any sequence preferences, except that the level of cleavage was lower (Figure 6A). Together, this data suggests that unlike Exo1, which is a structure-specific nuclease, the *Tetrahymena *nuclease is a sequence-dependent exonuclease.

## Discussion

We have identified a sequence-dependent exonuclease activity that possesses the biochemical properties to process C-rich telomere strands and generate 3' G-strand overhangs. This 3'-overhang is a ubiquitous structure of eukaryotic telomeres, and is essential for telomere end protection and for elongation by telomerase. The *Tetrahymena *nuclease we described is a 100 kDa enzyme that shows preference for cleaving AA- or CA-ending residues that are present in the C-strand of telomeric repeats.

The existence of a telomere-specific nuclease has been postulated for years. A number of nucleases including Mre11, Sae2, Dna2, Artemis, Apollo, Werner syndrome protein (WRN), and Exo1 have been examined for their role in different aspects of telomere processing [37,43-52]. Mre11 and WRN are both 3'-5' nucleases that have been found associated with telomeres [53-57]. While the directionality of Mre11 and WRN is not consistent with 5'-strand resection at the telomere, recent biochemical studies of WRN show a sequence-specific cleavage on telomeric 3'-overhang [46], suggesting WRN may pay a role in processing the G-rich overhang strand.

Sae2, Artemis, Apollo, and Dna2 are 5' nucleases, but none of these nucleases show biochemical specificity for either blunt-ended or telomeric substrates. Apollo, interacts with the telomere-binding protein, TRF2, and cleaves single-stranded DNA but shows no sequence preferences *in vitro *between telomeric repeats and unique sequences [58,59]. Recent studies have showed that Apollo is recruited by TRF2 and plays a role in processing leading-strand overhangs in mice [51], and human Apollo cooperates with TRF2 to help resolve topological stress during telomere replication [60]. Sae2 is an endonuclease with substrate specificity on 3' overhangs, hairpins, and branched DNA [61], and it processes hairpin structures and meiotic double-strand breaks together with MRX/N complex [61-66]. Dna2 is a multi-functional enzyme that possesses helicase and single-stranded DNA specific endonuclease activities. In *S. pombe*, Dna2 was implicated in telomere 3'-overhang generation [48], but *in vitro*, Dna2 shows no sequence specificity for cleavage of single-stranded DNA (Kao & Bambara, unpublished results). These differences in biological specificity indicate that the *Tetrahymena *exonuclease is not the same enzyme as any of these known 5'-3' nucleases.

Because our enzyme has a 5'-3' exonuclease activity, we compared its biochemical properties with Exo1. Exo1 is a well-characterized 5'-3' nuclease that plays a role in multiple DNA metabolic pathways [41], including telomere maintenance [44,49,52,67-69]. *In vivo *evidence suggests that Exo1 contributes to telomere maintenance [44] and may participate in the generation of ssDNA at dysfunctional telomeres [52,67]. Studies have also shown that Exo1 is important in processing ends for the recombination pathways that allow survivors to grow in the absence of telomerase in yeast [68]. The biochemical specificity we report for the *Tetrahymena *exonuclease is different from the properties of Exo1. The *Tetrahymena *nuclease has specificity for telomere repeats ending in 5'-AA and 5'-CA, but Exo 1 did not show sequence specificity. In addition, Exo1 shows much higher activity on phosphorylated substrates ([70], Figure 6, and Additional file 3, supplementary Figure S3) while the *Tetrahymena *exonuclease has significant activity on both unphosphorylated and phosphorylated substrates. These differences in specificity indicate that the *Tetrahymena *exonuclease that we purified is distinct from Exo1.

Several lines of evidence indicate that C-strand processing is regulated; the precise structure of 3'-overhangs in ciliates [14,16,28], the cell cycle-specific generation of 3'-overhangs in yeast, and the formation of overhangs in the absence of telomerase [26,27]. This regulation occurs through both Cdk1 [25,29] and through telomere-specific binding proteins. Telomere end-binding proteins, such as TEBP αβ heterodimer, first discovered in *Oxytricha *[71,72], bind to single-stranded telomeric DNA through an OB fold and protect the telomeres from degradation, recombination, and end-joining [73,74]. In yeast, Cdc13 binds to the single-stranded overhang, and together with Stn1 and Ten1 [75] protect the ends and play a role in end-processing and telomere elongation. In mammalian cells, Pot1, a component of the shelterin complex, binds the single-stranded overhang through an OB fold domain similar to the TEBP α [76]. Pot1 also plays a role along with other shelterin complex components in regulating access of processing nucleases to telomeres [29,77]. Thus, the specific action of telomere processing nucleases on telomeres will involve interaction with these telomere-specific binding proteins.

## Conclusions

Telomere structure and function is conserved throughout eukaryotes. Telomerase is present in most eukaryotes and is essential for telomere elongation. The negative feedback mechanism in which telomere-binding proteins inhibit elongation of long telomeres and allow the establishment of a telomere length equilibrium [22,78] is also conserved from yeast to mammals. While the sequence of telomere-binding proteins in different organisms differ, the function of these proteins in end protection and regulation of telomere elongation is highly conserved [79]. Given this conservation, we think it is likely that telomere processing is also conserved. While there are likely multiple factors involved in telomere processing, the sequence-specific properties of the *Tetrahymena *exonuclease described here suggest it may play a role in telomere processing.

## Methods

### *Tetrahymena *extracts

Both mated and unmated cell extracts were tested, and nuclease activity was found in both. Mated cell extracts were obtained as described previously [80]. Unmated cell extracts were obtained by growing 36 liters of B2086 *Tetrahymena *cells without starvation or mating steps.

### Nuclease Purification

All the purification steps for *Tetrahymena *nuclease were carried out at 4°C. Approximately 1 liter of total cell extracts was first fractionated by (NH_4_)_2_SO_4 _precipitation. The supernatant was collected, and the pellets were resuspended in TMG^+ ^buffer (TMG: 10 mM Tris, pH 8.0, 1 mM MgCl_2_, 10% glycerol, 10 mM β-mercaptoethanol, 0.1 mM PMSF, with RNase inhibitor and 1× protease inhibitor cocktail). Nuclease activity was detected in pellets of 60% and 70% fractions, which were combined and loaded onto a Phenyl Sepharose column on an ÄKTA Fast Protein Liquid Chromatography system, FPLC (GE Healthcare, Amersham Biosciences, NJ). Proteins were eluted at a gradient of TMG-1.7 M (NH_4_)_2_SO_4 _to TMG-0 M. Nuclease activity was detected in TMG 400 mM ~ 500 mM (NH_4_)_2_SO_4_. The positive fractions were pooled, dialyzed, and then loaded onto an Affi-Gel Blue Gel column (Bio-Rad, CA). The proteins were eluted in a gradient from TMG-0M KCl to TMG-1M KCl, and nuclease activity was detected in TMG 200 mM ~ 400 mM KCl fractions. The positive fractions were combined, dialyzed, and loaded onto a Mono-Q column (GE Healthcare, Amersham Biosciences, NJ). The proteins were eluted with a TMG-0M KCl to TMG-1M KCl gradient, and active fractions eluted at TMG 250 mM KCl ~ 400 mM KCl. The nuclease activity positive fractions were combined, concentrated with a Q spin column (Viva Science, Germany), and loaded onto a Superdex 200 (GE Healthcare, Amersham Biosciences, NJ) gel filtration column with TMG-100 mM KCl buffer. A gel filtration standard (Bio-Rad, CA) was used to estimate the MW of the nuclease. Kav was calculated by the following equation: Kav = (Ve-Vo)/(Vt-Vo), where Kav is a constant that is between 0-1, Ve is elution volume for the protein, Vo is column void volume (typically, 30% of the total column volume), and Vt is total bed volume. Kav was then plotted against Log MW of the standard, which was used to determine the MW of the nuclease. The nuclease ran at approximately 100 KDa (Figure 1D). Active fractions were combined and loaded onto a hydroxyapatite, HAP, column (Bio-Rad, CA). The column was eluted with a gradient from TMG-0M to TMG-250 mM potassium phosphate, and the active fractions were found at 15 ~ 60 mM potassium phosphate. ~ 2,500 fold enriched nuclease preparation was obtained, and the active and purified fractions were combined, dialyzed, and concentrated using a Mono-Q or Q spin column.

### Oligonucleotide substrates

The oligonucleotide sequences utilized in this report are listed in Additional file 4, supplementary Table S1. Oligonucleotides were annealed to generate double-stranded substrates as described in the figure legends. A schematic representation of substrates is shown in Figure 1A and subsequent figures. Each internally-labeled oligonucleotide substrate was generated by labeling the downstream primers using [γ-^32^P]ATP (6000 Ci/mmol) (PerkinElmer Life Science Products, MA) and T4 polynucleotide kinase (Roche Applied Sciences, IN), followed by ligation. End-labeled substrates were obtained by using [γ-^32^P]ATP (6000 Ci/mmol) and T4 polynucleotide kinase. The biotinylated substrates and unmodified primers were synthesized by Operon Biotechnologies, Inc. (Huntsville, AL), and the RNA-containing and phosphorothioate oligonucleotides were from Integrated DNA Technology (Coralville, IA). The 10-nucleotide markers were obtained from GIBCO (Invitrogen-GIBCO, CA). All radiolabeled primers were purified by gel isolation from 10% polyacrylamide, 7 M urea, denaturing gels.

### Nuclease assays

Activity assays were performed in reaction buffer that was adapted from the telomerase assay conditions [24]: 50 mM Tris-HCl (pH 8.0), 5 mM β-mercaptoethanol, 50 mM KCl, and 10 mM MgCl_2_, 1 mM spermindine, and 0.1 μl of RNase inhibitor. Exo1 activity was done in a reaction buffer condition adapted as described previously [41]: 20 mM Hepes (pH 7.8), 50 mM KCl, 0.5 mM DTT, 5 mM MgCl_2_, 0.05% Triton X-100, 100 ug/mL BSA, and 5% glycerol. Each reaction contained 5 fmol substrate and 25-fold excess of streptavidin in a 20-μl reaction volume with different amounts of purified nuclease as indicated in the figure legends. All the assays were preincubated at 30°C for 5 min. to allow streptavidin and biotin binding (Additional file 1, supplementary Figure S1). The reactions were initiated at 30°C or 37°C (for hExo1) for 15 or 20 min., and reactions were then stopped by the addition of 20 μl 2× termination dye (95% formamide (v/v) with bromophenol blue and xylene cyanol). The heat-denatured reactions were resolved on either 10% or 15% polyacrylamide, 7 M urea denaturing gels. Each gel was quantitated using a PhosphorImager (GE Healthcare, NJ) and analyzed using ImageQuant v1.2 software from Molecular Dynamics (GE Healthcare, NJ). 10-nucleotide markers are run and labeled on the left side of all shown gels. All assays were performed multiple times, and representative assays are shown.

## Authors' contributions

HKT designed research, performed experiments, analyzed data, and wrote the paper. CWG designed research, analyzed data, and wrote the paper. Both authors read and approved the final manuscript.

## Supplementary Material

Additional file 1 **Supplementary Figure S1 - Majority of biotinylated substrates were streptavidin bound**. A gel-shift assay is shown in 10% native polyacrylamide gel. Substrates in reaction buffers (conditions as described in "Methods") were incubated with and without streptavidin for 5 min. at 30°C. The mixtures were in 1× DNA loading dye (0.04% bromophenol blue, 0.04% xylene cyanol, and 5% glycerol) at a final volume of 12 μl containing a total of 5 fmol substrates. Half of this mixture was loaded onto a 10% native gel. The gel was then dried and viewed by PhosphorImager (GE Healthcare, NJ). *Lanes 1*, *3*, *5*, *7*, *9*, *11*, *13*, *15*, *17*, *19*, *21*, and *23 *are substrates without streptavidin, and *lanes 2*, *4*, *6*, *8*, *10*, *12*, *14*, *16*, *18*, *20*, *22*, and *24 *are substrates with streptavidin. Schematic representations of substrates are shown on top of the gel, and the substrates utilized (from left to right) are: Ia, Ib, IIa, IIb, IIIa, IIIb, IVa, IVb, VIa, VIb, Va, and Vb, respectively. Click here for file

Additional file 2 **Supplementary Figure S2 - Protein gel and enzymatic assay of fractions from final Mono-Q concentration step are shown**. (A) A gradient 8-14% SDS-PAGE was performed, followed by silver-staining (Invitrogen, CA). Fractions were labeled on top of the gel, and two different markers were run and labeled on either side of the gel. "F" indicates fraction, and "W" refers to salt washes. (B) A 10% urea denaturing polyacrylamide gel was utilized. Enzymatic reactions of fractions found in (A) are shown. Click here for file

Additional file 3 **Supplementary Figure S3 - Unlike Exo1, *Tetrahymena *nuclease cleavage is more sequence-dependent**. Various permutations of telomeric repeats that are with and without 5'-phosphorylation is shown (20 min reactions on a 10% urea gel). The substrates (left to right) are: S1:S18*, S3:S20*, S9:S26*, S10:S27*, S11:S28*, S12:S29*, and S13:S30*. Click here for file

Additional file 4 **Supplementary Table S1**. This file contains a list of all the oligonucleotides used in this study. Click here for file
