# Supplementary figures and images for: A sequence-dependent exonuclease activity from Tetrahymena thermophila
Source: BMC Biochem. 2010 Nov 16;11:45. doi: 10.1186/1471-2091-11-45 (PMC2998447; doi:10.1186/1471-2091-11-45)

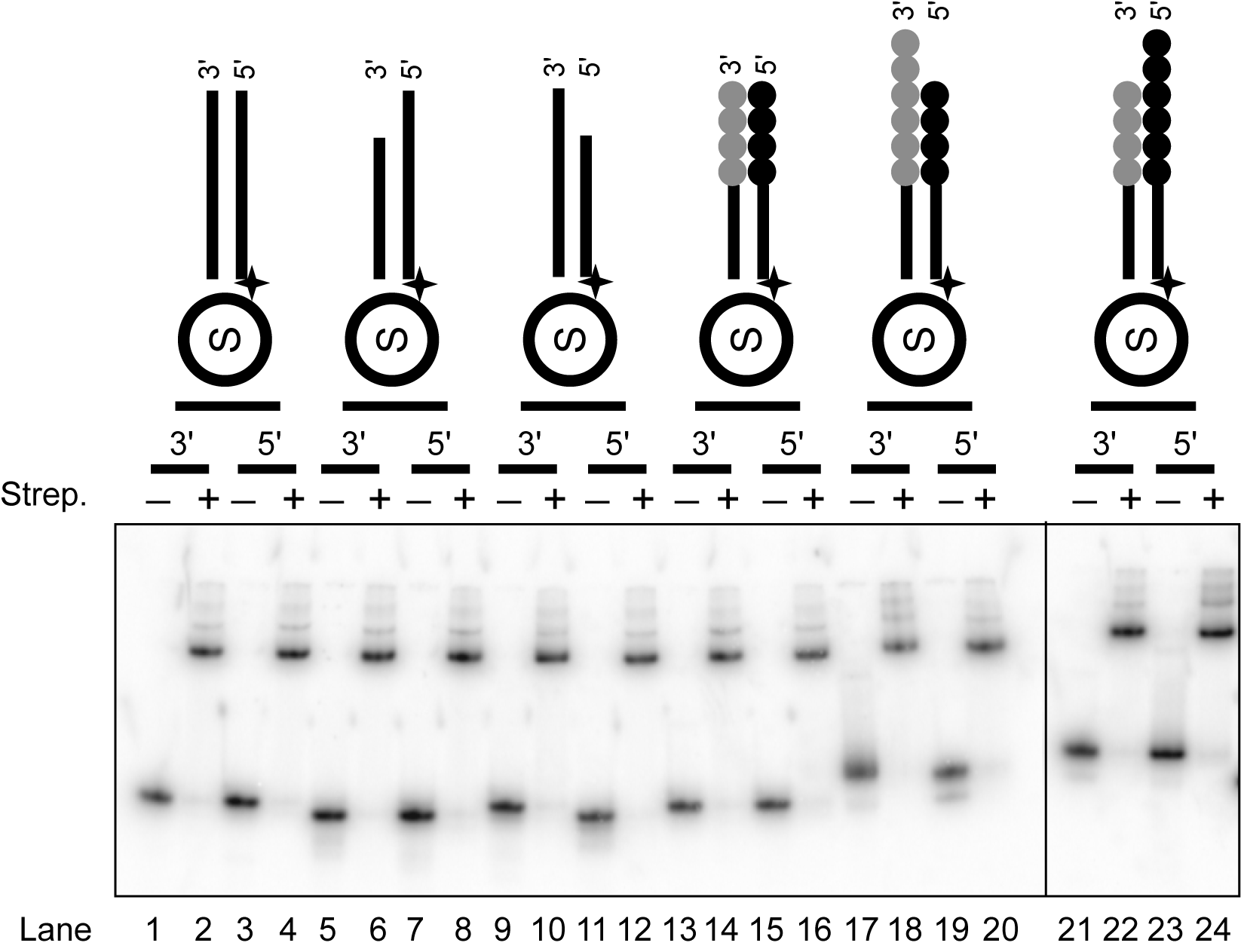

Supplement: Additional file 1 — Supplementary Figure S1 - Majority of biotinylated substrates were streptavidin bound. A gel-shift assay is shown in 10% native polyacrylamide gel. Substrates in reaction buffers (conditions as described in "Methods") were incubated with and without streptavidin for 5 min. at 30°C. The mixtures were in 1× DNA loading dye (0.04% bromophenol blue, 0.04% xylene cyanol, and 5% glycerol) at a final volume of 12 μl containing a total of 5 fmol substrates. Half of this mixture was loaded onto a 10% native gel. The gel was then dried and viewed by PhosphorImager (GE Healthcare, NJ). Lanes 1, 3, 5, 7, 9, 11, 13, 15, 17, 19, 21, and 23 are substrates without streptavidin, and lanes 2, 4, 6, 8, 10, 12, 14, 16, 18, 20, 22, and 24 are substrates with streptavidin. Schematic representations of substrates are shown on top of the gel, and the substrates utilized (from left to right) are: Ia, Ib, IIa, IIb, IIIa, IIIb, IVa, IVb, VIa, VIb, Va, and Vb, respectively. [file 1471-2091-11-45-S1.TIFF]

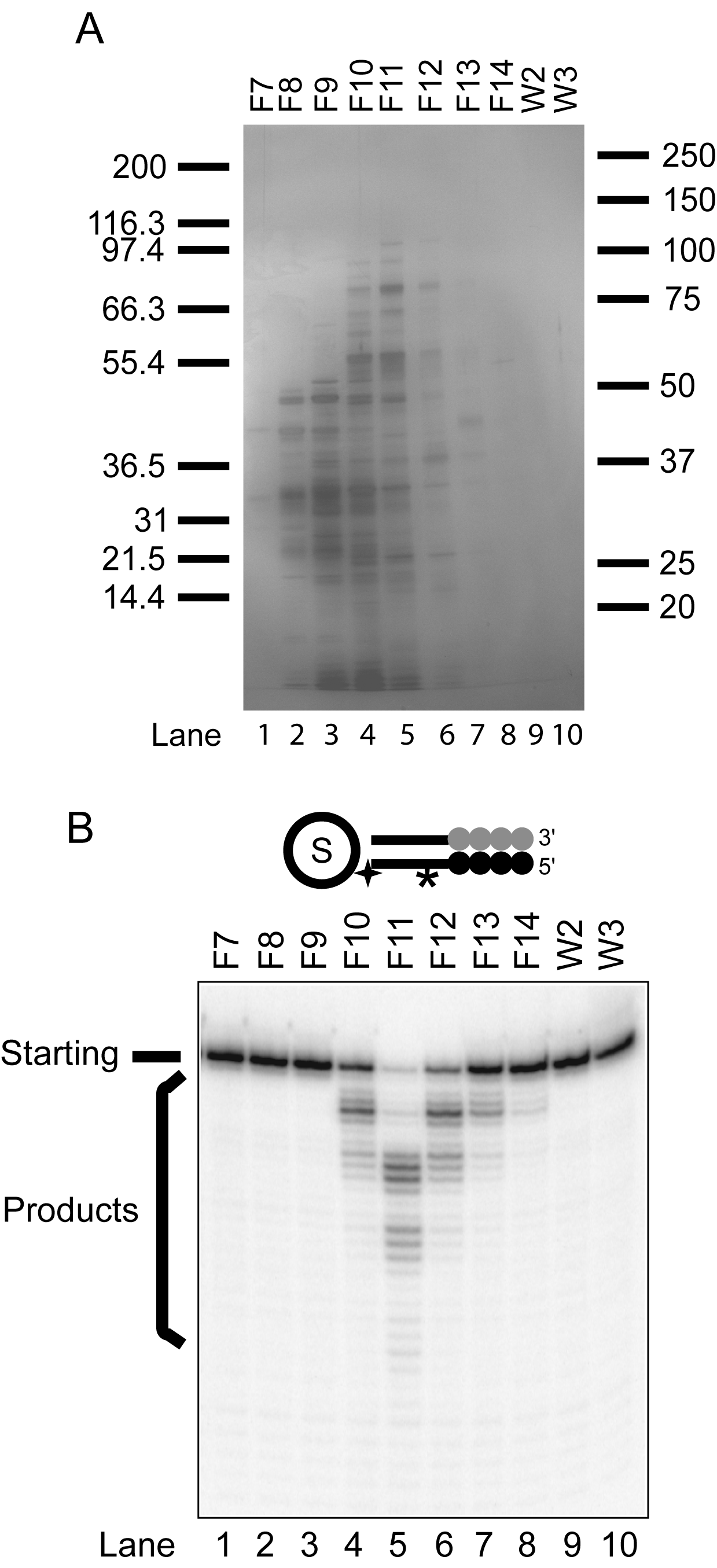

Supplement: Additional file 2 — Supplementary Figure S2 - Protein gel and enzymatic assay of fractions from final Mono-Q concentration step are shown. (A) A gradient 8-14% SDS-PAGE was performed, followed by silver-staining (Invitrogen, CA). Fractions were labeled on top of the gel, and two different markers were run and labeled on either side of the gel. "F" indicates fraction, and "W" refers to salt washes. (B) A 10% urea denaturing polyacrylamide gel was utilized. Enzymatic reactions of fractions found in (A) are shown. [file 1471-2091-11-45-S2.TIFF]

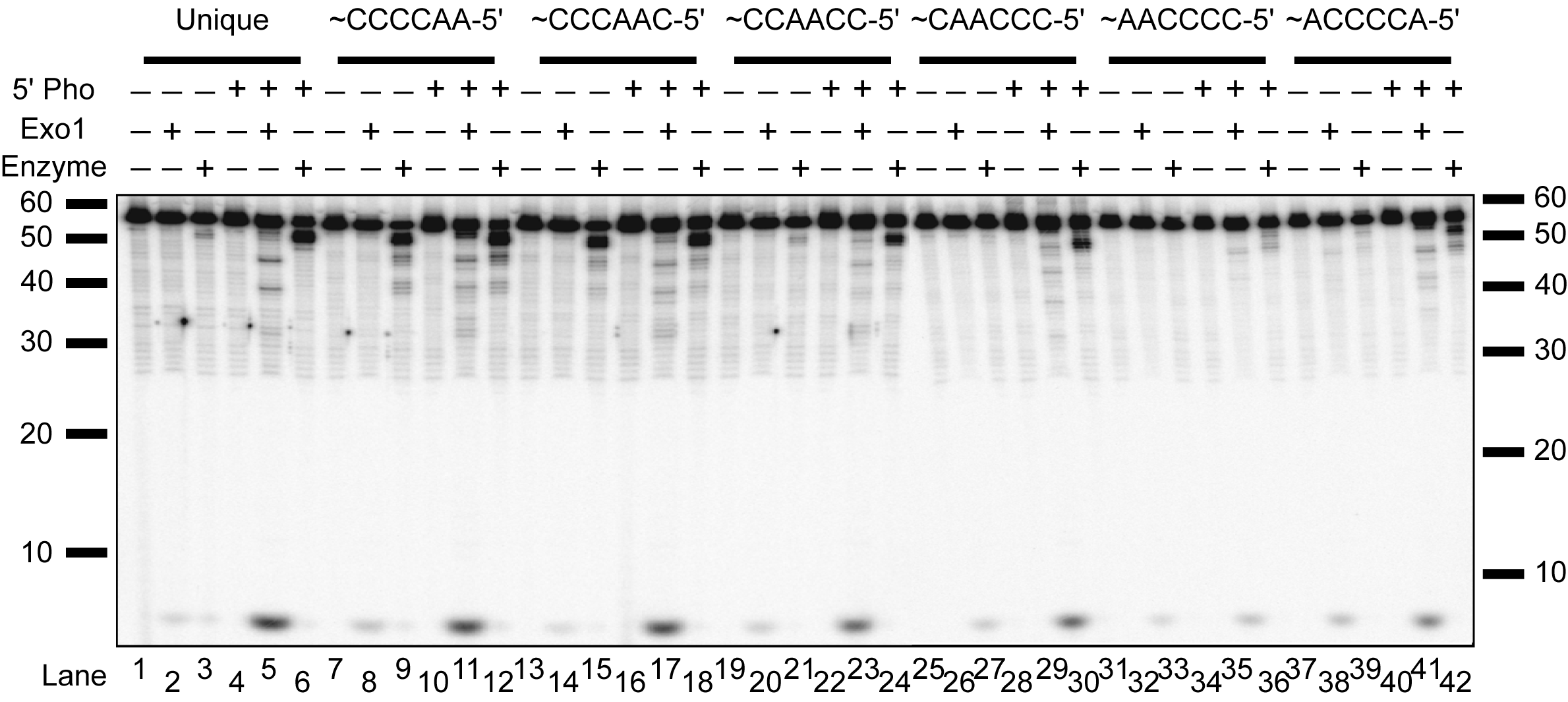

Supplement: Additional file 3 — Supplementary Figure S3 - Unlike Exo1, Tetrahymena nuclease cleavage is more sequence-dependent. Various permutations of telomeric repeats that are with and without 5'-phosphorylation is shown (20 min reactions on a 10% urea gel). The substrates (left to right) are: S1:S18*, S3:S20*, S9:S26*, S10:S27*, S11:S28*, S12:S29*, and S13:S30*. [file 1471-2091-11-45-S3.TIFF]
