# Supplementary material for: A sequence-dependent exonuclease activity from Tetrahymena thermophila
Source: BMC Biochem. 2010 Nov 16;11:45. doi: 10.1186/1471-2091-11-45 (PMC2998447; doi:10.1186/1471-2091-11-45)
Supplement: Additional file 4 — Supplementary Table S1. This file contains a list of all the oligonucleotides used in this study. [file 1471-2091-11-45-S4.DOC]

# TABLE S1. Oligonucleotide sequences (5'-3')

# _________________________________________________________________________________________________________________________­­­­­­­­­­­____________________________________________________

Unmodified Sequences*^a,b^*

S1 (52-mer) CCACCCGTCCACCCGACGCCAC*CTCCTGGTACCGAGGTCCAATCGCCGTTCG

S2 (40-mer) CCACCCGTCCACCCGACGCCAC*CTCCTGGTACCGAGGTCC

S3 (52-mer) CCACCCGTCCACCCGACGCCAC*CTCCTGGGGGTTGGGGTTGGGGTTGGGGTT

S4 (64-mer) CCACCCGTCCACCCGACGCCAC*CTCCTGGGGGTTGGGGTTGGGGTTGGGGTTGGGGTTGGGGTT

S5 (52-mer) CCACCCGTCCACCCGACGCCAC*CTCCTGCCCCAACCCCAACCCCAACCCCAA

S6 (52-mer) CCACCCGTCCACCCGACGCCAC*CTCCTGGGTTGGGGTTGGGGTTGGGGTTGG

S7 (52-mer) CCACCCGTCCACCCGACGCCAC*CTCCTGCCAACCCCAACCCCAACCCCAACC

S8 (52-mer) CCACCCGTCCACCCGACGCCAC*CTCCTGGACGATGACGATGACGATGACGAT

S9 (52-mer) CCACCCGTCCACCCGACGCCAC*CTCCTGGGGTTGGGGTTGGGGTTGGGGTTG

S10 (52-mer) CCACCCGTCCACCCGACGCCAC*CTCCTGGGTTGGGGTTGGGGTTGGGGTTGG

S11 (52-mer) CCACCCGTCCACCCGACGCCAC*CTCCTGGTTGGGGTTGGGGTTGGGGTTGGG

S12 (52-mer) CCACCCGTCCACCCGACGCCAC*CTCCTGTTGGGGTTGGGGTTGGGGTTGGGG

S13 (52-mer) CCACCCGTCCACCCGACGCCAC*CTCCTGTGGGGTTGGGGTTGGGGTTGGGGT

S14 (52-mer) CCACCCGTCCACCCGACGCCAC*CTCCTGAGGGTTAGGGTTAGGGTTAGGGTT

S15 (52-mer) CCACCCGTCCACCCGACGCCAC*CTCCTGTGGTGTGTGTGTGGGTGTGTGTGT

S16 (46-mer) CCACCCGTCCACCCGACGCCAC*CTCCTGGGGGTTGGGGTTGGGGTT

S17 (58-mer) CCACCCGTCCACCCGACGCCAC*CTCCTGGGGGTTGGGGTTGGGGTTGGGGTTGGGGTT

S18 (52-mer) CGAACGGCGATTGGACCTCGGTACCAGGAG*GTGGCGTCGGGTGGACGGGTGG

S19 (40-mer) GGACCTCGGTACCAGGAG*GTGGCGTCGGGTGGACGGGTGG

S20 (52-mer) AACCCCAACCCCAACCCCAACCCCCAGGAG*GTGGCGTCGGGTGGACGGGTGG

S21 (64-mer) AACCCCAACCCCAACCCCAACCCCAACCCCAACCCCCAGGAG*GTGGCGTCGGGTGGACGGGTGG

S22 (52-mer) TTGGGGTTGGGGTTGGGGTTGGGGCAGGAG*GTGGCGTCGGGTGGACGGGTGG

S23 (52-mer) CCAACCCCAACCCCAACCCCAACCCAGGAG*GTGGCGTCGGGTGGACGGGTGG

S24 (52-mer) GGTTGGGGTTGGGGTTGGGGTTGGCAGGAG*GTGGCGTCGGGTGGACGGGTGG

S25 (52-mer) ATCGTCATCGTCATCGTCATCGTCCAGGAG*GTGGCGTCGGGTGGACGGGTGG

S26 (52-mer) CAACCCCAACCCCAACCCCAACCCCAGGAG*GTGGCGTCGGGTGGACGGGTGG

S27 (52-mer) CCAACCCCAACCCCAACCCCAACCCAGGAG*GTGGCGTCGGGTGGACGGGTGG

S28 (52-mer) CCCAACCCCAACCCCAACCCCAACCAGGAG*GTGGCGTCGGGTGGACGGGTGG

S29 (52-mer) CCCCAACCCCAACCCCAACCCCAACAGGAG*GTGGCGTCGGGTGGACGGGTGG

S30 (52-mer) ACCCCAACCCCAACCCCAACCCCACAGGAG*GTGGCGTCGGGTGGACGGGTGG

S31 (52-mer) AACCCTAACCCTAACCCTAACCCTCAGGAG*GTGGCGTCGGGTGGACGGGTGG

S32 (52-mer) ACACACACACCCACACACACACCACAGGAG*GTGGCGTCGGGTGGACGGGTGG

S33 (46-mer) AACCCCAACCCCAACCCCCAGGAG*GTGGCGTCGGGTGGACGGGTGG

S34 (58-mer) AACCCCAACCCCAACCCCAACCCCAACCCCCAGGAG*GTGGCGTCGGGTGGACGGGTGG

S35 (52-mer) CCACCCGTCCACCCGACGCCAC*CTCCTGGTACCGAGGTCCAATCGCCGTTGT

S36 (52-mer) CCACCCGTCCACCCGACGCCAC*CTCCTGGTACCGAGGTCCAATCGCCGTTAT

S37 (52-mer) CCACCCGTCCACCCGACGCCAC*CTCCTGGTACCGAGGTCCAATCGCCGTTTT

S38 (52-mer) CCACCCGTCCACCCGACGCCAC*CTCCTGGTACCGAGGTCCAATCGCCGTTCT

S39 (52-mer) CCACCCGTCCACCCGACGCCAC*CTCCTGGTACCGAGGTCCAATCGCCGTTTG

S40 (52-mer) CCACCCGTCCACCCGACGCCAC*CTCCTGGTACCGAGGTCCAATCGCCGTTGG

S41 (52-mer) CCACCCGTCCACCCGACGCCAC*CTCCTGGTACCGAGGTCCAATCGCCGTTAG

S42 (52-mer) CCACCCGTCCACCCGACGCCAC*CTCCTGGTACCGAGGTCCAATCGCCGTTGA

S43 (52-mer) CCACCCGTCCACCCGACGCCAC*CTCCTGGTACCGAGGTCCAATCGCCGTTAA

S44 (52-mer) CCACCCGTCCACCCGACGCCAC*CTCCTGGTACCGAGGTCCAATCGCCGTTTA

S45 (52-mer) CCACCCGTCCACCCGACGCCAC*CTCCTGGTACCGAGGTCCAATCGCCGTTCA

S46 (52-mer) CCACCCGTCCACCCGACGCCAC*CTCCTGGTACCGAGGTCCAATCGCCGTTGC

S47 (52-mer) CCACCCGTCCACCCGACGCCAC*CTCCTGGTACCGAGGTCCAATCGCCGTTAC

S48 (52-mer) CCACCCGTCCACCCGACGCCAC*CTCCTGGTACCGAGGTCCAATCGCCGTTTC

S49 (52-mer) CCACCCGTCCACCCGACGCCAC*CTCCTGGTACCGAGGTCCAATCGCCGTTCC

S50 (52-mer) CCACCCGTCCACCCGACGCCAC*CTCCTGGTACCGAGGTCCAATCGCCGCAGT

S51 (52-mer) CCACCCGTCCACCCGACGCCAC*CTCCTGGTACCGAGGTCCAATCGCCGCAAT

S52 (52-mer) CCACCCGTCCACCCGACGCCAC*CTCCTGGTACCGAGGTCCAATCGCCGCATT

S53 (52-mer) CCACCCGTCCACCCGACGCCAC*CTCCTGGTACCGAGGTCCAATCGCCGCACT

S54 (58-mer) CCACCCGTCCACCCGACGCCAC*CTCCTGGTACCGAGGTCCAATCGCCGTTCGCTACGC

S55 (46-mer) CCACCCGTCCACCCGACGCCAC*CTCCTGGTACCGAGGTCCAATCGC

S56 (58-mer) CCACCCGTCCACCCGACGCCAC*CTCCTGGTACCGAGGTCCAATCGCCGCATTCTACGC

S57 (52-mer) ACAACGGCGATTGGACCTCGGTACCAGGAG*GTGGCGTCGGGTGGACGGGTGG

S58 (52-mer) ATAACGGCGATTGGACCTCGGTACCAGGAG*GTGGCGTCGGGTGGACGGGTGG

S59 (52-mer) AAAACGGCGATTGGACCTCGGTACCAGGAG*GTGGCGTCGGGTGGACGGGTGG

S60 (52-mer) AGAACGGCGATTGGACCTCGGTACCAGGAG*GTGGCGTCGGGTGGACGGGTGG

S61 (52-mer) CAAACGGCGATTGGACCTCGGTACCAGGAG*GTGGCGTCGGGTGGACGGGTGG

S62 (52-mer) CCAACGGCGATTGGACCTCGGTACCAGGAG*GTGGCGTCGGGTGGACGGGTGG

S63 (52-mer) CTAACGGCGATTGGACCTCGGTACCAGGAG*GTGGCGTCGGGTGGACGGGTGG

S64 (52-mer) TCAACGGCGATTGGACCTCGGTACCAGGAG*GTGGCGTCGGGTGGACGGGTGG

S65 (52-mer) TTAACGGCGATTGGACCTCGGTACCAGGAG*GTGGCGTCGGGTGGACGGGTGG

S66 (52-mer) TAAACGGCGATTGGACCTCGGTACCAGGAG*GTGGCGTCGGGTGGACGGGTGG

S67 (52-mer) TGAACGGCGATTGGACCTCGGTACCAGGAG*GTGGCGTCGGGTGGACGGGTGG

S68 (52-mer) GCAACGGCGATTGGACCTCGGTACCAGGAG*GTGGCGTCGGGTGGACGGGTGG

S69 (52-mer) GTAACGGCGATTGGACCTCGGTACCAGGAG*GTGGCGTCGGGTGGACGGGTGG

S70 (52-mer) GAAACGGCGATTGGACCTCGGTACCAGGAG*GTGGCGTCGGGTGGACGGGTGG

S71 (52-mer) GGAACGGCGATTGGACCTCGGTACCAGGAG*GTGGCGTCGGGTGGACGGGTGG

S72 (52-mer) ACTGCGGCGATTGGACCTCGGTACCAGGAG*GTGGCGTCGGGTGGACGGGTGG

S73 (52-mer) ATTGCGGCGATTGGACCTCGGTACCAGGAG*GTGGCGTCGGGTGGACGGGTGG

S74 (52-mer) AATGCGGCGATTGGACCTCGGTACCAGGAG*GTGGCGTCGGGTGGACGGGTGG

S75 (52-mer) AGTGCGGCGATTGGACCTCGGTACCAGGAG*GTGGCGTCGGGTGGACGGGTGG

Modified Sequences*^a,b,c^*

M1 (52-mer) CCACCCGTCCACCCGACGCCAC*CTCCTGrGrUrArCrCrGrArGrGrUrCrCrArArUrCrGrCrCrGrUrUrCrG

M2 (52-mer) CCACCCGTCCACCCGACGCCAC*CTCCTGrGrGrGrGrUrUrGrGrGrGrUrUrGrGrGrGrUrUrGrGrGrGrUrU

M3 (52-mer) rCrGrArArCrGrGrCrGrArUrUrGrGrArCrCrUrCrGrGrUrArCCAGGAG*GTGGCGTCGGGTGGACGGGTGG

M4 (52-mer) rArArCrCrCrCrArArCrCrCrCrArArCrCrCrCrArArCrCrCrCCAGGAG*GTGGCGTCGGGTGGACGGGTGG

_____________________________________________________________________________________________________________________________________________________________________________

*^a^*The “*” sign indicates the location of the internally-label site for radiation P^32^. *^b^*The underlined nucleotide represents the location of a biotin modification on an labeled primer. *^c^*The nucleotide with a lower case “r” is a ribonucleotide.
